# The Effect of Mother's Gentle Human Touch on Preterm Neonate's Pain and Maternal Anxiety During Venipuncture in Turkiye

**DOI:** 10.1002/nur.22472

**Published:** 2025-05-20

**Authors:** Derya Kılınç, Seda Çağlar

**Affiliations:** ^1^ Department of Pediatric Nursing University of Health Science, Hamidiye Faculty of Nursing Istanbul Turkiye; ^2^ Department of Pediatric Nursing Istanbul University‐Cerrahpaşa, Florence Nightingale Faculty of Nursing Istanbul Turkiye

**Keywords:** anxiety, Gentle Human Touch, neonatal nursing, pain, preterm infant, venipuncture

## Abstract

Pain in neonates causes many negative effects, such as decreased oxygenation, deterioration in hemodynamics, and increased intracranial pressure. Mothers may experience anxiety while observing the infant experiencing pain. The aim of this study was to determine the effect of gentle human touch (GHT), applied by mothers, on the infant's physiological and pain responses during venipuncture, the mother's associated anxiety levels, and the correlation between infant pain and maternal anxiety. A randomized controlled study was conducted with 80 healthy preterm infants (40 GHT and 40 control) being seen in the neonatal follow‐up clinic 24–48 h post discharge from the hospital in Turkiye. Infants were 32–37 weeks gestational age. Infant pain was rated with the Neonatal Infant Pain Scale (NIPS), oxygen saturation and heart rate (HR) were taken from clinic monitors, length of crying was measured in minutes with a stop watch, and maternal anxiety was assessed with the State‐Trait Anxiety Inventory (STAI‐I). Infants in the GHT group had higher oxygen saturation values, lower pain scores, and shorter crying duration, but higher peak heart rates. Their mothers had lower anxiety scores. Maternal anxiety was strongly correlated with infant pain levels. The GHT method applied by mothers during venipuncture was shown to be effective in reducing infant pain, regulating infant physiological parameters, and reducing maternal anxiety.

**Trial Registration:** NCT05727631

## 1 Introduction

An average of 15 million babies are born preterm each year before the 37th week, and this number is increasing rapidly (Ohuma et al. 2023). During routine care of neonates, some painful invasive procedures are usually inevitable for both healthy and sick neonates. Due to their need for diagnostic and therapeutic measures, these infants frequently undergo painful and invasive procedures such as venipuncture (Cruz et al. 2016). Acute pain in preterm infants caused by invasive interventions has many negative impacts both physiologically and behaviorally in the short and long term such as reduced oxygenation, hemodynamic instability, increased intracranial pressure, anxiety, hyperalgesia, restlessness, sleep disturbance, malnutrition in the short term, and long term effects of delayed immune function, emotional disturbance, hyperactivity and attention deficit that would be manifest later in the child's life (Askarinia, Razban, Nematollahi and Mangolian Shahrbabaki 2024; Grunau 2020; McPherson et al. 2020).

## 2 Background

In recent years, Nonpharmacological methods have been used for pain management in neonates due to the lack of side effects (Stadler and Raith 2018). Frequently used Nonpharmacological methods such as skin‐to‐skin contact, swaddling, breastfeeding, aromatherapy, music therapy, massage, and touching are reported to lower anxiety scores, regulate physiological parameters and increase comfort (Benoit et al. 2017; Çamur and Erdoğan 2022; Moore et al. 2016; Shukla et al. 2018; Usta, Tanyeri‐Bayraktar and Bayraktar 2021). During painful interventions, the presence of parents, and participation of the parent in the procedure were effective for pain management, and this was reported to reduce anxiety experienced by the family (Azak et al. 2022; Eissler et al. 2022; Jones et al. 2021).

Touch is one of the earliest senses to develop during fetal development and remains one of the most developed senses after birth. This early development increases the sensitivity of newborns, especially preterm infants, to tactile stimuli and ensures that appropriately provided touch has positive effects on neurological and psychosocial development. Touch is also one of the developmental care methods used to reduce pain and calm the baby. (Humphrey 1964). When touch is used sensitively, it nurtures an infant's basic feeling of trust and positively affects psychosocial and physical development (Can and Kaya 2022; Dur et al. 2020; Sun et al. 2020; Bucsea and Pillai Riddell 2019). Touch therapy is a noninvasive treatment that does not require special equipment and technology and can be easily performed. It can also reduce the cost of treatment, length of illness, and complications (Fatollahzade et al. 2022). Although massage is a touch therapy know to reduce pain (Hauck et al. 2024), massage is not feasible during invasive procedures. Gentle human touch (GHT), another of the therapeutic types of touch, is a sensitive touch stimulant applied to the skin, ensuring a type of comfort without massage (Çağlar et al. 2025). The mother takes a deep breath and gathers Qi energy in the palm of her hand. Qi, a concept in traditional Chinese medicine, is considered an intangible life force that is thought to manifest through electromagnetic fields. In this technique, one hand is placed on the newborn's head while the other is placed on the abdomen to promote a calming effect. With this technique, one hand is placed on the head of the neonate and the other is placed on the abdomen to ensure a sedative effect. The GHT method is thought to reduce the experience of pain by distracting the infant, thus reducing conduction of pain stimuli to the cerebral cortex (Bembich et al. 2018). Studies researching the effects of GHT for preterm neonates have shown that it is effective in reducing procedural pain and stabilizing physiological parameters (Harrison et al. 1996; Dur et al. 2020; Fadlalmola et al. 2023; Fatollahzade et al. 2022; Sezer Efe et al. 2022).

Family‐centered care (FCC) supports family integration in line with individualized care principles, encouraging active parental participation in medical procedures involving their infant (Olsson et al. 2025). FCC promotes weight gain in preterm infants, shortens hospital stays, calms the infant during invasive procedures, supports healthy neurological development, and strengthens the mother‐infant bond, making the family an integral part of the caregiving process (Olsson et al. 2025; Vittner, Butler, Lawhon and Buehler 2025). Therefore, in this study, the GHT technique was applied by the mother during intravenous procedures, and its effects on maternal anxiety and infant pain were evaluated. Although there is insufficient data on the effects of maternal GHT during painful procedures on infants, the effects of Yakson or Gentle Human Touch methods applied by mothers to their preterm newborns on mother‐infant attachment, infant physiological responses, and length of hospital stay were exemined without ezamining infants’ pain and mothers’ anxiety levels (Can and Kaya 2022). In their 2015 study, Kastral et al. examined the effect of skin‐to‐skin contact (kangaroo care), in which the baby is placed directly on the mother's bare chest, on dyadic salivary cortisol levels, during heel prick blood collection from preterm neonates. Results indicated that kangaroo care decreased the pain score of the infants and a weak relationship was found between the cortisol levels of the mother and the infant.

Nonpharmacological pain management is one of the most important responsibilities of neonatal nurses. Encouraging parental participation during painful procedures has been found to be important. However, no studies of the effects on both infant and mother of GHT administered by mothers during the routine painful procedure of venipuncture were found in the literature. Therefore, the purpose of this study was to determine the effects of GHT applied by mothers during venipuncture on (1) the infant's physiological responses (HR, oxygen saturation), crying, and pain scores. and (2) maternal anxiety. The correlation between infant pain scores and maternal anxiety also was examined.

### 2.1 Research Hypotheses

The hypotheses were:

A. Preterm neonates whose mothers applied GHT during venipuncture compared to those whose mothers did not would show:

1. More favorable physiological responses, including lower HR and higher oxygen saturation
2. Shorter crying time
3. Lower pain scores

B. Mothers who applied GHT would show lower anxiety levels than mothers who did not.

C. Infant pain scores would be correlated with maternal anxiety scores.

## 3 Methods

### 3.1 Study Design

**Figure 1:**
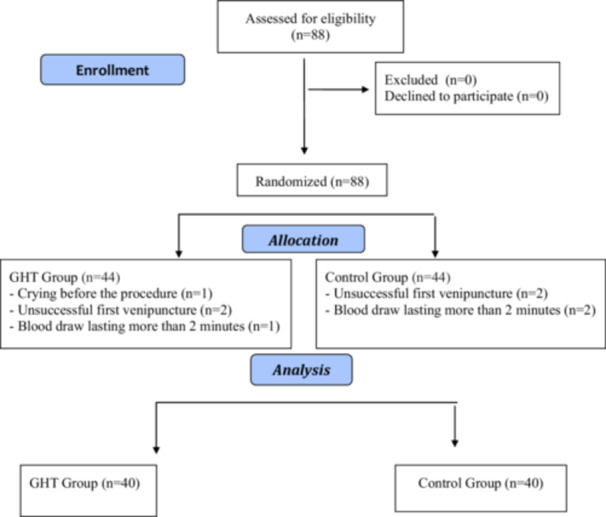
CONSORT flow diagram of the study.

This study was in an experimental, parallel‐group (intervention‐control), and randomized controlled design. The study design and implementation were based on the principles in the CONSORT list (Consolidated Standards of Reporting Trials) (Figure 1) (Benoit et al. 2017).

### 3.2 Study Setting

Data for this prospective, randomized controlled trial were collected in the blood collection room of the neonatal outpatient clinic of a teaching and research hospital between June 2021 and December 2021.

#### 3.2.1 Participants

The gestational age of the preterm neonates in the study was 32‐37 weeks. Mothers of preterm neonates were invited to participate in the study when they brought their infants for their outpatient appointments within 24 to 48 h post dicharge (around 33 weeks gestational age) and according to the order of admission to the neonatal outpatient clinic. Infants in the sample had to be deemed healthy by the clinic staff, and had to be calm and not crying before the blood draw. Mothers who agreed to participate had to speak and understand Turkish, and have no communication problems such as hearing impairment. The mothers agreed to participate in the study through an informed consent form given in the neonatal outpatient clinic before blood collection. Infants were excluded if they had previous blood draws, unsuccessful first blood draw attempts, collection procedures lasting longer than 2 min, had painkillers administered less than 6 h before the procedure, had congenital anomalies, or had been in the neonatal intensive care unit. Additionally, the infants of mothers in the control group who touched their babies during blood collection were excluded from the study.

To calculate the sample size, a power analysis was performed based on NIPS mean pain scores in a similar study by Sun et al. (2020) with 80% power and a margin of error of 0.05. Thus, 88 neonates were required to detect a [large effect] of GHT on infant pain scores with [99% power] power and alpha = 0.05. Thus, we planned to include a total of 88 preterm neonates in the research (GHT group=44, control group=44).

#### 3.2.2 Data Collection Tools

For collection of research data, the Descriptive Information Form, Neonatal Infant Pain Scale (NIPS) and State‐Trait Anxiety Inventory‐I (STAI‐I) were used.

##### Demographics

This form included a total of 8 items related to information about the preterm neonates (gestational week, birth weight, postnatal age, gender, type of birth, heart rate, oxygen saturation level and crying duration).

##### 3.2.2.1 Pain

Neonatal Infant Pain Scale (NIPS), was used to assess infant pain status (Sezer Efe et al. 2022; Lawrence, Alcock, Kay and McGrath 1991). NIPS is a pain scale including five types of behavior and respiration including facial expression, cry, breathing pattern, arms, legs, and state of arousal. While points of 0‐2 are given for crying, points of 0–1 are given for behavior. Total scores range from 0 to 7. High scores indicate increased pain severity. The NIPS had Cronbach alpha values from 0.83 to 0.86 (Sezer Efe et al. 2022). In the current study, the Cronbach alpha values for the scale before, during and after the procedure were 0.89, 0.83 and 0.92, respectively. The researcher and one independent nurse observer trained in coding behavioral state rated the NIPS concurrently. The before, during, and after interrater reliability was assessed with cohen kappas of 0.317, 0.705, and 1.000, respectively, and observer agreement of r = 0.88–0.99; *p* < 0.001, using observer average scores.

##### Maternal Anxiety

The anxiety section of the State‐Trait Anxiety Inventory‐I (STAI‐I; Spielberger et al. 2017) was used to assess the mothers’ anxiety expressed as feelings related to the situation they are in by describing how they feel at a certain moment and under certain conditions. After being informed which group they were in, the mothers were asked to complete the anxiety section of the STAI‐I. The scale had Cronbach alpha values from 0.83 to 0.87 (Spielberger et al. 2017). In the current study, the Cronbach alpha values before, during and after the procedure were 0.83, 0.87 and 0.84.

#### 3.2.3 Equipment

Oxygen saturation and heart rate measurements of preterm neonates were assessed using the, Covidien brand Nellcor model, MBB1508482 serial number, calibrated (3262‐0722‐16864) pulseoximeter device.

#### 3.2.4 Infant Crying

A Samsung Note‐10‐Plus model phone stopwatch was used to determine the crying time of preterm neonates. During the procedure, the stopwatch was turned on when the baby cried, and the stopwatch was stopped when the crying ended.

### 3.3 Ethical Considerations

The study adhered to the guidelines established in the Declaration of Helsinki. Before starting the research, ethical approval was obtained from the Ethics Committee of the XX X), and institutional permission was granted by the YY (Y). The study was registered in the Clinical Trials system (XX). Mothers were informed that they could withdraw from the study at any time and that their information would not be shared with anyone other than the researchers. Informed consent was obtained from mothers who agreed to participate in the study through the “Informed Voluntary Consent Form”.

#### 3.3.1 Interventions

##### Intervention Group

**Figure 2:**
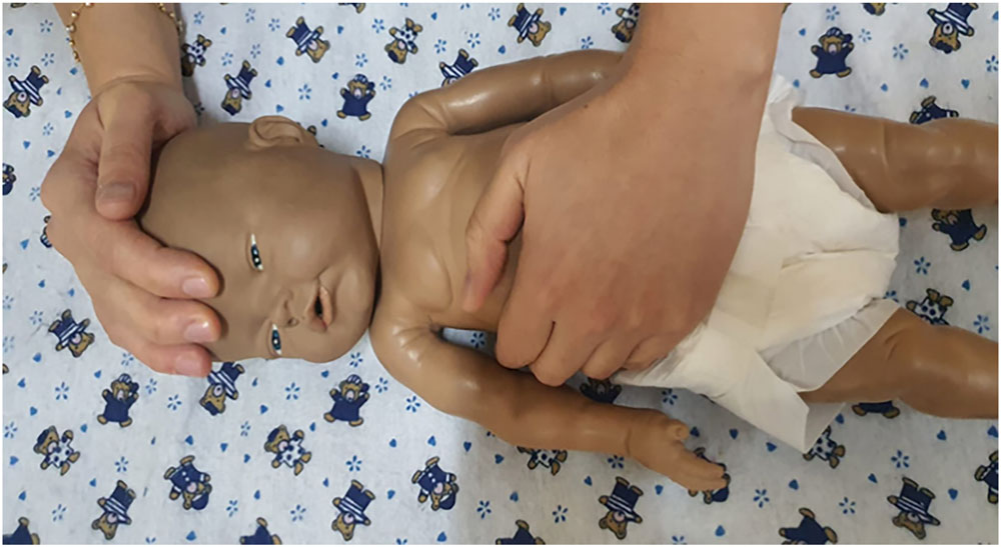
GHT method.

The mothers in the intervention were informed about GHT technique by the researcher. Mothers in the GHT groups touched their babies with the GHT technique during blood collection. Mothers were given gowns and masks before the procedure. They washed their hands and warmed them with a radiant heater to palm temperature of 34 degrees Celsius measured with a contact‐free thermometer. Mothers took a deep breath and waited a minute to accumulate “Qi” energy in their hands. They placed the palm of one hand on the infant's crown and the palm of the other hand on the lower abdomen encompassing the waist and hips and only touched the infant without massaging (Figure 2). The mothers touched the infants with the GHT technique for 5 min before blood collection and for 10 min during and after blood collection for a total of 15 min.

##### Control Group

Preterm neonates in the control group underwent the standard procedure used in the clinic. Mothers stayed in the same environment as the infants. Mothers were not advised about touching their infants.

### 3.4 Procedures

Infant participants were assigned study identification numbers from 1 to 88 using a simple computerized randomization method (randomizer. org). The mothers included in the study were assigned to the GHT or control groups by opaque sealed envelope method. Assignment to the GHT group and control groups was made by an independent statistician. The mothers drew a card from an opaque envelope in order of presentation to the blood collection outpatient clinic to determine which group they would be assigned to. After being informed which group they were in, the mothers were asked to complete the STAI‐I state anxiety inventory. Blinding was not possible because the investigators and participants were aware of the intervention.

After receiving instructions, mothers in both groups completed the STAI‐I scale 5 min before the procedure, immediately after the procedure and at 15 min after the procedure. The STAI scale was administered after explaining which group they would be in, as it was thought that it could help mothers understand the procedure better and that being with their baby and touching her during this process could reduce their anxiety levels before the procedure. 5 min before the procedure, in the first minute of the procedure and fifth minute after the procedure, the SpO_2_, HR and NIPS pain scores of the infants and crying duration during the procedure were recorded on the data collection form.

Infants in all groups had blood collection completed under a radiant heater in supine position, from the left hand with no. 21 needle by the researcher nurse with 14 years of pediatric nursing experience.

In line with the routine procedure in the blood collection room of the clinic, infants and mothers in both groups listened to Johannes Brahms‐Wiegenlid Op. 49 No.4 lullaby (45 decibel). Mothers of infants in both groups were taken to the blood collection room and were beside their infants during the procedure.

### 3.5 Statistical Analysis

Analysis of research data used the licensed SPSS 28.0 program. Analysis of descriptive statistics for data used mean, standard deviation, median, minimum, maximum, percentage value, and interclass correlation for compatibility analysis. Distribution of variables was measured with the Kolmogorov Smirnov test. The Mann‐Whitney U test was used for analysis of quantitative independent data. The Wilcoxon test was used for analysis of dependent quantitative data. For analysis of categorical independent data, the chi‐square test was used or the Fisher test was used if chi‐square test conditions were not met. For correlation analysis, the Spearman test was used.

## 4 Results

During the data collection process, a total of 8 infants were excluded from the research due to crying before blood collection (*n* = 1), unsuccessful first blood collection attempt (*n* = 2), and blood collection lasting more than 2 min (*n* = 1) in the GHT group and due to unsuccessful first blood collection attempt (*n* = 2) and blood collection lasting more than 2 min (*n* = 2) in the control group. The final number of infant participants was 80 preterm neonates (GHT group=40, control group=40) (Figure 1).

The descriptive features of this sample of preterm neonates were similar between groups (gestational week, birth weight, postnatal age, gender, type of birth; see Table 1).

**Table 1:** Baseline characteristics of preterm neonates.

Characteristics		GHT Group (*n* = 40)	Control Group (*n* = 40)	Total	Test	*p*
**Gestational wk**	**(X̅** ± **SD) (Min‐Max)**	35.2 ± 1.4 33.3–36.0	34.8 ± 1.6 33.1–36.7	35.0 ± 1.5 34.0–36.0	*z* = 0.982	0.326
**Birth weight (g)**	**(X̅** ± **SD)**	2.519 ± 418	2.655 ± 445	2587 ± 434	*z* = 1.290	0.197
**Postnatal age (day)**	**(X̅** ± **SD)**	2.72 ± 0.59	2.57 ± 0.50	2.6 ± 0.55	*z* = 0.145	0.780
**Gender**	Female	22 (55%)	22 (55%)	44 (55.0%)	x²= 0.000	1.000
	Male	18 (45.0%)	18 (45.0%)	36 (45.0%)		
**Type of birth**	NSD	21 (52.5%)	17 (42.5%)	38 (47.5%)	x²= 0.802	0.370
	C/S	19 (47.5%)	23 (57.5%)	42 (52.5%)		
**Mother Age**	**(X̅** ± **SD)**	30.3 ± 6.4	29.0 ± 6.7	29.6 ± 6.6	*z* = 0.887	0.375

*Note:* %: row percentage, x̅: mean, SD: Standard Deviation ^z^ Mann–Whitney U test/^w^Wilcoxon test/^X²^ chi square test. NSD: Normal Spontaneous Delivery, C/S: Cesarean Section

### 4.1 Physiological Parameters (HR, Oxygen Saturation) of Preterm Neonates

Before blood collection, the mean peak HR for neonates were similar between the groups (*z* = 3.134, *p* > 0.05). During and after the procedure, the mean peak heart rate in the GHT group was lower (*z* = 1.640, *p* < 0.05 and *z* = 0.328, *p* < 0.05, respectively). Before blood collection, the mean oxygen saturation were similar in both groups (*z* = 2.626, *p* = 0.646). During and after the procedure, the oxygen saturation values in the GHT group were higher (*z* = 3.332, *p* < 0.05 and *z* = 2.453, *p* < 0.05, respectively). (see Table 2). The results of this study support Hypothesis A1, demonstrating that preterm neonates whose mothers applied GHT during venipuncture exhibited significantly more favorable physiological responses, including lower heart rate and higher oxygen saturation, compared to those whose mothers did not apply GHT.

**Table 2:** Physiological parameters and crying duration of preterm neonates.

Physiological parameters	GHT group (*n* = 40)	Control group (*n* = 40)	Test	*p*
**Heart rate (per minute) (x̅ ± SD)** Before procedure	133.6 ± 6.1	133.2 ± 7.6	*z* = 3.134	0.611
During procedure	135.4 ± 6.1	145.8 ± 8.6	*z* = 1.640	0.000
After procedure	134.1 ± 5.2	138.8 ± 7.1	*z* = 0.328	0.001
Before/during procedure	1.8 ± 3.5	12.6 ± 5.3	*z* = 7.260	0.000
In‐group p	0.005^w^	0.000^w^		
Before/after procedure	0.4 ± 3.8	5.6 ± 6.6	*z* = 4.474	0.000
In‐group p	0.716^w^	0.000^w^		
**Oxygen saturation (x̅ ± SD)** Before procedure	99.6 ± 0.5	99.7 ± 0.5	*z* = 2.626	0.646
During procedure	99.5 ± 0.6	98.3 ± 1.5	*z* = 3.332	0.000
After procedure	99.5 ± 0.6	99.8 ± 0.4	*z* = 2.453	0.049
Before/during procedure	−0.2 ± 0.5	−1.4 ± 1.4	*z* = −4.866	0.000
In‐group p	0.058^w^	0.000^w^		
Before/after procedure	0.1 ± 0.2	0.0 ± 0.4	*z* = 0.354	0.724
In‐group p	0.155^w^	0.157^w^		
**Crying time (minutes) (x̅±SD)**	6.3 ± 5.4	55.5 ± 12.4	*z* = −7.709	0.000

^z^Mann–Whitney U Test/^w^Wilcoxon

### 4.2 Crying Times of Preterm Neonates

The crying time durations in the GHT group were shorter compared to the crying durations in the control group (*z* = −7.709, *p* < 0.001) (see Table 2). The result of this study support Hypothesis A2, demonstrating that preterm neonates whose mothers applied GHT during venipuncture exhibited significantly shorter crying time.

### 4.3 Pain Scores of Preterm Neonates

Before blood collection, the pain scores for infants were similar between the groups (*z* = 0.310, *p* > 0.05). During and after the procedure, pain scores for infants in the GHT group were lower (*z* = −0.790, *p* < 0.05; and *z* = −0.167, *p* < 0.05, respectively) (see Table 3). The result of this study support Hypothesis A3, demonstrating that preterm neonates whose mothers applied GHT during venipuncture exhibited significantly lower pain scores.

**Table 3:** Pain scores of preterm neonates.

NIPS scores (x̅±SS)	GHT group (*n* = 40)	Control group (*n* = 40)	Test	*p*
Before procedure	0.35 ± 0.53	0.33 ± 0.47	*z* = 0.310	0.939
During procedure	1.45 ± 0.88	5.85 ± 1.05	*z* = −0.790	0.000
After procedure	0.08 ± 0.27	2.45 ± 0.81	*z* = −0.167	0.000
Before/during procedure	1.10 ± 0.74	5.53 ± 0.99	*z* = −0.803	0.000
In‐group p	0.000^w^	0.000^w^		
Before/after procedure	−0.28 ± 0.55	2.13 ± 0.91	*z* = −0.811	0.724
In‐group p	0.005^w^	0.000^w^		

^z^Mann–Whitney U Test/^w^Wilcoxon Test/x̅ mean

### 4.4 Levels of Maternal Anxiety

Before, during and after blood collection, the anxiety scores for mothers in the GHT group were lower (*z* = −6.128, *p* < 0.05; z = −7.300, *p* < 0.05; and z = −7.766, *p* < 0.05, respectively) (see Table 4). The result of this study support Hypothesis B, demonstrating that mothers who applied GHT showed lower anxiety levels than mothers who did not.

**Table 4:** Anxiety scores of mothers.

STAI scores (x̅±SS)	GHT group (*n* = 40)	Control group (*n* = 40)	Test	*p*
Before procedure	41.1 ± 10.0	57.8 ± 8.7	*z* = −6.128	0.000
During procedure	30.0 ± 7.0	70.3 ± 11.2	*z* = −7.300	0.000
After procedure	20.9 ± 1.3	35.4 ± 6.3	*z* = −7.766	0.000
Before/during procedure	−11.1 ± 9.1	12.5 ± 14.0	*z* = −6.822	0.000
In‐group p	0.000^w^	0.000^w^		
Before/after procedure	−20.3 ± 9.8	−22.3 ± 9.7	*z* = −1.006	0.314
In‐group p	0.000^w^	0.000^w^		

^z^Mann–Whitney U Test/^w^Wilcoxon Test/x̅ mean

### 4.5 Correlation Between Infant Pain Scores and Maternal Anxiety Levels

There were positive significant correlations between the anxiety scores of mothers and pain scores of preterm neonates before (*r* = 0.543, *p* < 0.001), during (*r* = 0.736, *p* < 0.001) and after (*p* = 0.786, *p* < 0.001) blood collection (*p* < 0.05) (see Table 5). The result of this study support Hypothesis C, demonstrating that infant pain scores correlated with maternal anxiety scores.

**Table 5:** Correlation between preterm neonates pain scores and mothers’ anxiety scores.

			Mothers' anxiety scores		
			Before procedure	During procedure	After procedure
**Infants’ Pain Scores**	Before procedure	rho	−0.200	−0.048	0.031
		*p*	0.075	0.673	0.785
	During procedure	rho	0.543	0.736	0.786
		*p*	0.000*	0.000*	0.000*
	After procedure	rho	0.555	0.705	0.773
		*p*	0.000*	0.000*	0.000*

*Note:* Spearman correlation test, rho: correlation coefficient

## 5 Discussion

This study is the first to examine the effect of the gentle human touch (GHT) method applied by mothers to preterm neonates during blood collection on (1) physiological parameters (peak HR, saturation), pain scores (NIPS), and crying durations of infants and on (2) maternal anxiety (STAI‐I) as well as the relationship between preterm infant pain and mothers’ anxiety.

In this study, HR were lower, oxygen saturation values were higher, crying durations were shorter, and pain scores were lower in preterm neonates in the GHT group. The anxiety scores were lower in mothers in the GHT group. There was a strong positive correlation between the anxiety scores of mothers and the pain scores of preterm neonates.

The GHT method has been shown to result in more oxygen being sent to the brain by stimulating blood circulation, improving cardiac performance, and calming infants between, 32–37 weeks of gestational age (Sezer Efe et al. 2022). Similarly, in the study by Fatollahzade et al. (2022), the GHT method applied during endotracheal aspiration of preterm neonates was found to positively affect heart rate, respiratory rate and crying durations of infants (Fatollahzade et al. (2022). Çağlar et al. (2025) reported that the Yakson and GHT methods applied during endotracheal aspiration reduced HR while increasing oxygen saturation levels compared to infants in the control group, among preterm infants between 26 and 36 weeks of gestational age (Çağlar et al. 2025). Riadini et al. (2019) concluded that the GHT method had positive impacts on oxygen saturation of preterm neonates. A meta‐analysis study by Fadlalmola et al. (2023) found that, unlike our study, the GHT method had no effect on HR and oxygen saturation in preterm neonates (Fadlalmola et al. 2023). This difference is thought to arise from the variation in the type of invasive procedures applied, such as those used by Sun et al. (2020) during retinopathy of prematurity screening and by Sezer Efe during heel lancing. In the previous noted studies, nurses applied the GHT method. We found only one study where mothers applied the GHT method to preterm neonates (Can ve Kaya 2022). In this study, Can and Kaya determined that the GHT method had positive impacts on mother‐baby bonding, behavioral status of the infant (sleep and calmness), respiratory rate, HR, duration of hospitalization and physical development.

The sense of touch begins to develop in the antenatal period and is strengthened by skin‐to‐skin contact between infant and mother after birth (Can and Kaya 2022; Karakaş 2019). Touch develops the bond between mother and infant, while also reducing stress and anxiety (Can and Kaya 2022; Höbek Akarsu et al. 2017; Çağlayan et al. 2015). GHT is a therapeutic type of touch and is one of the nonpharmacological pain‐relieving methods. Touching the infant with light pressure ensures the infant's comfort by surface stimulation of nerve endings in the skin surface. Nerves in the skin send messages to the brain through the neural network in the spinal cord. This process essentially stimulates pressure receptors and activates the parasympathetic nervous system (Varela et al. 2018). It has been found that GHT applied to preterm neonates reduces the infant's reaction to pain by increasing β‐endorphin levels (Qiu et al. 2017). Research on GHT has consistently demonstrated pain reduction in preterm neonates who were undergoing medical procedures.

Similar to our study results, GHT has been used to manage pain in preterm neonates during screening for retinopathy of prematurity (Sun et al. 2020), tracheal aspiration (Fatollahzade et al. 2022; Oliveira et al. 2023), heel sticks (Dur et al. 2020; Sezer Efe et al. 2022), and other methods of blood collection (Bahrami et al. 2023).

There are studies about the importance of the presence of parents during interventional procedures on preventing pain and anxiety (Azak et al. 2022; Sağlık and Çağlar 2019; Filippa et al. 2021; Rad et al. 2021). In our research, the mean anxiety scores for mothers using the GHT method on their neonates were lower 5 min before, during the procedure, immediately after the procedure and at 15 min after the procedure. The low anxiety scores for mothers before the procedure are thought to be due to having been given information about the GHT method. There were strong and positive correlations between mean anxiety scores for mothers and mean pain scores for preterm neonates (*p* < 0.05).

A study by Özarslan et al. (2024) found that as mothers of infants in the neonatal intensive care unit received family‐centered care, their anxiety levels decreased. This finding, similar to our research, demonstrates that family‐centered care practices positively impact mothers’ psychological well‐being.

In Turkey, social contact is considered an expression of closeness and sincerity, with touch therapy playing a significant role, especially in the mother‐infant relationship. Immediately after birth, skin‐to‐skin contact with the mother is established, and breastfeeding within the first hour is encouraged. This practice strengthens the mother‐infant bond, stimulates the infant sense of touch, and positively effects on the infant's development. In our study, it was hypothesized that the mother's application of Gentle Human Touch (GHT) might be related to the tradition of touch in Turkish culture, as it reduced both the mother's anxiety score and the preterm infant's pain score. However, since there is a lack of research on the role and effects of touch in Turkish culture in the literature, this issue could not be addressed. In this sense, our study will make a significant contribution to the existing literature.

## 6 Practice Implications

Preterm infants are frequently exposed to painful procedures for diagnostic and treatment purposes. These painful interventions can negatively affect their development. Today, there is strong evidence supporting the use of nonpharmacological methods to reduce the adverse effects of painful procedures. The GHT technique stands out as an effective pain management method, as it does not require any equipment, special technology, or extensive training. As demonstrated in our study, the use of the GHT technique by mothers during painful procedures effectively reduces both maternal anxiety levels and infants’ pain scores. Therefore, nurses working in blood collection rooms should implement the GHT technique while actively involving mothers in the process.

In line with these results, neonatal nurses should be informed about the importance of therapeutic touch and applying touch methods. The nonpharmacological pain relief method of GHT should be used as standard procedure during painful procedures in neonatal clinics. Mothers should be with their infants during procedures while informing them about the GHT method and allowing them to touch their infants. The use of family supported care is recommended to reduce maternal anxiety during painful interventions in preterm neonates.

### 6.1 Limitations

This study has both strengths and limitations. Among its strengths, it comprehensively evaluates preterm infants’ pain levels, physiological parameters (HR, oxygen saturation, crying duration), and maternal anxiety scores before, during, and after the intravenous procedure. Additionally, the reliability of the data was enhanced by having the NIPS pain scores assessed by two independent observers. This study is the first in the literature to examine the effect of the GHT method applied by mothers during intravenous blood collection on the pain scores of preterm infants. Additionally, it is the first study to evaluate the correlation between preterm infants’ pain levels and mothers’ STAI‐I anxiety scale scores during an intravenous procedure, making a significant contribution to the literature. The application of the STAI‐I anxiety scale after the mothers’ group assignments were determined and the researcher's awareness of the group assignments are limitations of the study. Data were only collected from a single center and cannot be generalized. Because infants were excluded if they required more than one attempt at venipuncture and for procedures lasting more than 2 min, that effects of GHT for repeated attempts of invasive procedures and for more difficult blood collections are unknown.

## 7 Conclusion

The findings of this study demonstrate that the GHT method applied by mothers during venipuncture is an effective approach for regulating the physiological parameters of preterm neonates, reducing their crying duration and pain scores, and lowering maternal anxiety. Additionally, a strong correlation was found between maternal anxiety and preterm neonates' pain levels.

The effects of the GHT method applied by mothers on maternal and infant health needs further investigation, including randomized controlled trials comparing the GHT method with different pain relief methods so that we can ensure that nursing pain relief interventions are providing optimal support for these vulnerable infants and their mothers.

## Author Contributions

Seda Çağlar. and Derya Kılınç conceptualized and designed the study: Seda Çağlar. administrated the project and data collection: Derya Kılınç coordinated and supervised data collection: Seda Çağlar. and Derya Kılınç performed data analysis: Derya Kılınç drafted the initial manuscript: and Seda Çağlar. critically reviewed and revised the manuscript. All the authors read and approved the final version of the manuscript.

## Ethics Statement

Ethical approval was provided by the Zeynep Kamil Women and Children Diseases Traning and Research Hospital Clinical Research Ethics Committee (Date: 09.12.2020, Number: 190), and institutional permission were obtained before beginning the study (Date: 23.02.2021, Number: 2021‐06). Written informed consent was obtained from the infants’ parents.

## Conflicts of Interest

The authors declare no conflicts of interest.

## Data Availability

The authors have nothing to report.
